# Advanced wound healing in a patient with transmetatarsal amputation caused by severe diabetic foot infection: A case report

**DOI:** 10.1016/j.ijscr.2023.109180

**Published:** 2024-01-10

**Authors:** V.R. Serrudo, R. Saurral, R. Pool, A. Kruler, N. Sanchez, L.M. Carrio

**Affiliations:** Hospital Municipal de Trauma y Emergencias Dr Federico Abete, Centro Municipal de Diabetes Dr. Alberto Maggio, Hospital de Día de Pie Diabético “Polo Sanitario”, República Argentina

**Keywords:** Case report, Diabetic foot, Wound healing, Biofim, Silver sulfadiazine

## Abstract

Introduction and importance: Diabetic foot accounts for 50% to 95 % of non-traumatic amputations. The healing process of a surgical wound resulting from amputation in the diabetic foot is complex, and it is difficult to achieve an optimal outcome, which should include obtaining a functional stump for the patient. Healing is mainly hindered by infection, vascular disease, and wound size. In turn, biofilm formation significantly delays the healing process, increasing morbidity and impairing the amputee's quality of life. Case presentation: This study analyzes the case of an 80-year-old male patient with diabetes who had failed to respond to previous treatment on an infected wound from a transmetatarsal amputation. The new treatment involved spraying the wound with silver sulfadiazine, lidocaine, and vitamin A aerosol and covering it with gauze dressings soaked in silver sulfadiazine, lidocaine, and vitamin A. The case evolution indicators used were total wound area, percentage of granulation tissue, wound perimeter, and maximum distance between the wound edges. A 3D simulation was also used to assess the wound bed. Clinical Discussion: Biofilm is linked to slower wound healing and wound chronicity, as this community of microorganisms in the wound slows down healing even when there are no apparent signs of infection. Therefore, treatment should be geared toward preventing contamination from leading to biofilm formation. Conclusion: Our results show that silver sulfadiazine, lidocaine, vitamin A gauze dressings, and aerosol have promoted fast and effective healing in a diabetic patient with a wound at high risk of greater amputation.

## Highlights

• Rigorous treatment of the surgical wound resulting from an amputation should be considered
• Control biofilm formation
• Avoid new amputation
• The use of gauze and spray with silver sulfadiazine, lidocaine, and vitamin A significantly improved wound progression
• A gauze dressings and aerosol helped achieve a fast and optimal evolution of the wound

## 1 Introduction

It is estimated that 15 % to 25 % of patients with diabetes mellitus develop foot ulcers at some point in their lives. In general, these ulcers precede total or partial amputation. A study in Argentina [1] showed that diabetic foot is the reason for hospitalization in 3.16 % of total patients and 17.85 % of diabetic patients. Moreover, diabetic foot accounts for 50 % to 95 % of non-traumatic amputations [2,3].

In a retrospective review of outcomes [4] performed on patients who underwent transmetatarsal amputation at a single United Kingdom hospital between 2005 and 2017, it was shown that a 78 % healing rate had been achieved, with a median healing time of 83 days and a median duration of hospital admission of 24 days. No further surgery to the same foot was required after the wounds healed. In another study [5], complete wound healing was reached in only 52 % of the patients within 12 months. The need for additional surgery or major amputation was 56 % and 30 %, respectively. The need for an additional procedure was particularly high after transmetatarsal amputation (64 %). Risk factors for non-healing or a major amputation were infection, ischemia, and a history of peripheral arterial occlusive disease.

The healing process of a surgical wound resulting from amputation in the diabetic foot is complex, and it is difficult to achieve an optimal outcome. Treatment should aim to achieve the least retraction possible so that the scar is functional and prevents further issues for the patient. The stump should be adequately shaped, functional, painless, and properly covered with tissue at the bone ends, as well as have good blood irrigation and an optimal dermal cover.

Method: This case report has been reported in line with the SCARE Criteria [6]. This article describes the use of silver sulfadiazine, lidocaine, and vitamin A gauze dressings and aerosol to treat an infected wound from a transmetatarsal amputation performed 3 months earlier on an 80-year-old male diabetic patient. We aimed to evaluate a potential therapeutic standard that can avoid adverse issues in amputation wounds by controlling contamination, biofilm formation, and pain while obtaining a functional stump that improves the patient's quality of life.

When the treatment was prescribed, the wound was not healing adequately. However, when the silver sulfadiazine, lidocaine, and vitamin A gauze dressings and aerosol were used, the wound area and the necrotic tissue quickly shrunk, the granulation tissue increased, and the distance between the edges reduced.

The case evolution indicators used were total wound area, percentage of granulation tissue, wound perimeter, and maximum distance between the wound edges. As a secondary goal, the patient's safety was assessed, including the nature and incidence of any adverse event.

Wound evolution was monitored with periodical photographs and data analysis using the ImageJ software from the National Institutes of Health (NIH), USA (Rasband WS, ImageJ U. S. National Institutes of Health, Bethesda, Maryland, USA, https://imagej.nih.gov/ij/, 1997–2018).

## 2 Narrative

Eighty-year-old male patient. Type 2 diabetic, insulin-independent with 9 years of disease evolution. Diabetic nerve disease, a peripheral vascular disease with no revascularization option, dyslipidemia. A previous minor amputation on the left foot was performed in 2018.

The patient's treatment regimen included cilostazol (100 mg every 12 h), rosuvastatin (20 mg), aspirin (100 mg), and clopidogrel (75 mg), insulin glargine and insulin aspart on a basal-bolus regimen. The mechanisms of action of these drugs are described as follows:

Cilostazol is a reversible phosphodiesterase 3 inhibitor which functions as an antiplatelet and vasodilator agent.

Rosuvastatin increases the number of hepatic LDL receptors on the cell surface to enhance uptake and catabolism of LDL. Additionally, it inhibits hepatic synthesis of VLDL, which reduces the total number of VLDL and LDL particles.

Aspirin irreversibly inhibits cyclooxygenase by acetylating its hydroxy group, thereby blocking thromboxane A(2) synthesis. A single 325-mg dose of aspirin is sufficient to achieve an almost 90 % inhibition rate.

Clopidogrel inhibits platelet aggregation in the same way as ticlopidine does. Its active metabolite selectively and irreversibly blocks ADP from binding to its platelet receptor and prevents the subsequent ADP-mediated activation of the glycoprotein GPIIb/IIIa complex.

In addition, the evolution of the wound was monitored in collaboration with the diabetic foot care team.

The patient came to the Hospital de Día de Pie Diabético at the Polo Sanitario of Los Polvorines, in Malvinas Argentinas, Province of Buenos Aires, Argentina, (Diagnostics Table 1, Timeline Table 2), on October 23, 2020, to see a doctor about necrosis on the fifth toe of the right foot. A biopsy was performed on the remaining bone, testing positive for *Pseudomonas aeruginosa*. Lab results came back with high inflammatory markers. A targeted antibiotic treatment was prescribed.

**Table 1:** Diagnostics.

Type	Value	Unit
17/10/2020		
ALP (alkaline phosphatase)	173	IU/L
ALT (alanine aminotransferase)	16	IU/L
AST (aspartate aminotransferase)	40	U/L
Cholesterol, HDL	47	mg/dL
Cholesterol, LDL	161	mg/dL
Cholesterol, total	226	mg/dL
Creatinine	1.25	mg/dL
CRP (C-reactive protein)	7	mg/dL
ESR (erythrocyte sedimentation rate)	18	mm/1st hour
Glucose	104	mg/dL
Hb (hemoglobin)	11.67	g/dL
HbA1C (hemoglobin A1C)	6.5	%
Hct (hematocrit)	39	%
Platelet count	193,000	cells/μL
Serum urea	36	mg/dL
Triglycerides	90	mg/dL

12/2/2021		
Creatinine	1.08	mg/dL
CRP (C-reactive protein)	48	mg/dL
CSF white blood cell count	15,000	cells/μL
ESR (erythrocyte sedimentation rate)	85	mm/1st hour
Glucose	118	mg/dL
Hb (hemoglobin)	11.8	g/dL
HbA1C (hemoglobin A1C)	6.6	%
Hct (hematocrit)	36.8	%
Serum urea	32	mg/dL

15/3/2021		
Creatinine	1.49	mg/dL
CRP (C-reactive protein)	12	mg/dL
CSF white blood cell count	8400	cells/μL
Glucose	119	mg/dL
Hb (hemoglobin)	95	g/dL
HbA1C (hemoglobin A1C)	6.5	%
Hct (hematocrit)	33.4	%
Platelet count	276.600	cells/μL
Serum urea	35	mg/dL

31/5/2021		
ALP (alkaline phosphatase)	202	IU/L
ALT (alanine aminotransferase)	14	IU/L
AST (aspartate aminotransferase)	45	IU/L
Cholesterol, HDL	40	mg/dL
Cholesterol, LDL	81	mg/dL
Creatinine	1.13	mg/dL
CRP (C-reactive protein)	12	mg/dL
CSF red blood cell count	9700	cells/μL
ESR (erythrocyte sedimentation rate)	10	mm/1st hour
Glucose	110	mg/dL
Hb (hemoglobin)	93	g/dL
HbA1C (hemoglobin A1C)	6	%
Hct (hematocrit)	311	%
Platelet count	359,900	cells/μL
Serum urea	23	mg/dL
Triglycerides	46	mg/dL

**Table 2:** Timeline.

22/10/2020	Consultation due to necrosis of the fifth toe, right foot, bone cannula, iodoformedgauze healing, bone bx requested, empirical treatment started VO AMC –TMS, laboratories requested, nutrition and education ic
3/11/2020	Poor evolution ATB is rotated to the DELABAXI protocol (delafloxacin 450 mg) PPS culture is taken and right foot X-ray control, management with 26 IU I Giargina sc C/24 h arterial Doppler of lower limbs is requested
15/2/2021	Arterial Doppler result on 02/16/21 right side tib before and after decreased velocities, monophasic, compatible with moderate obstructive compromise
22/2/2021	Negative pps culture results from 4/11/2020 AMC and TMS
	The patient's treatment regimen included cilostazol (100 mg every 12 h), rosuvastatin (20 mg), aspirin (100 mg), and clopidogrel (75 mg). In addition, the evolution of the wound was monitored in collaboration with the diabetic foot care team.
23/2/2021	Spontaneous amputation of the fifth finger, dry necrosis of the fourth toe on the right foot, healing of iodine-formed gauzes. RX right foot osteomyelitis, fourth and fifth toe
2/3/2021	Lower limb arteriography is requested, fourth, third and finger necrosis is delimited with alcohol, second finger with cyanosis alcohol is indicated
4/4/2021	Vascular surgery indicates
5/4/2021	Result of angiography performed on 30/3/2021 right side proximal occluded anterior tibial artery, occluded proximal peroneal artery, occluded posterior tibial artery.
5/4/2021	Left side proximal occluded anterior tibial artery, slow flow peroneal artery, occludes at the distal level, occluded posterior tibial artery, continuous cures with alcohol
5/4/2021	Angiography 30/3/2021 Right proximal occluded anterior tibial artery, occluded proximal fibular artery and posterior tibial artery. Left proximal tibial anterior artery occluded, peroneal slow flow, occluded distally, posterior tibial artery occluded. We immediately consulted with the vascular surgery area, where it was determined that revascularization was not a possibility in this case, given the moderate-to-severe nature of the stenosis.
21/4/2021	Digital necrosis delimited with alcohol, that day vascular surgery indicates digital amputation, IC with O and T from the Diabetic Foot team is requested. AMG 90–120 with symptoms of hypoglycemia is indicated I Giargin 20 IU
26/4/2021	Due to poor evolution and cyanosis of the hallux, transmetatarsal amputation was decided in the operating room, samples of the remaining bone were taken for culture.
28/4/2021	Postoperative control of transmetatarsal amputation with bleeding is indicated flat cure with alcohol control with O and T
28/5/2021	Positive bone biopsy result: *Citrobacter freundii*, Pseudomona aeruginosa, *Enterococcus faecalis*
28/5/2021	It presents with necrosis of the edges, distal points are removed, healing with iodoformed gauzes, necrosis of the lateral edges is observed, poor evolution
	Treatment of delafloxacin 450 mg iv every 12 h at home
8/6/2021	Transmetarsal amputation ulcer devitalized tissue in the amputation site bed, moderate to severe pain in treatment with Delafloxacin IV, Toilette and bone bx taken in the operating room by O and T. Healing with iodine-formed gauzes and collagenase
11/6/2021	Healing with pocket iodine-formed gauzes and granulating base with collagenase
15/6/2021	Amputation stump ulcer with abundant exudate and fibrin, iodine-formed gauzes indicated moderate pain at the time of healing, the appointment for control with alarm guidelines. Bone bx culture result received from 12/6/2021 IM penicillin is indicated
15/6/2021	Bone biopsy culture result received on 12/6/21 was positive for: *Pseudomona aeuriginosa*, *Enterococcus Faecalis*, *Escherichia Coli*
22/6/2021	Poor evolution and contamination of the wound. Cleaning with soap and water and treatment every 12 h at home with gauze soaked in Silver Sulfadiazine, Lidocaine and Vitamin A and Silver Sulfadiazine, Lidocaine and Vitamin A spray are indicated.
5/7/2021	He attends control with improvement in control of exudate, decrease in pain, mechanical debridement is performed with a continuous scalpel, cures with soaked gauzes and silver sulfadiazine spray, lidocaine, and vitamin A every 12 h.
2/8/2021	Concurs healing with marked improvement in pain and good evolution background of amputation stump ulcer with decreased fibrin, continuous healing at home with aerosol and gauze soaked in silver sulfadiazine, lidocaine, vitamin A
2/8/2021	Wound with good evolution. Lesion area: 164.42 cm^2^; perimeter: 52.17 cm; necrotic tissue area: 52.01 %; Granulation area percentage: 31.6 %
16/9/2021	Clopidogrel 75 mg every 24 h is added, amputation of stump ulcer with fibrinogranulating bottom, diameter decrease and pain control, continuous healing at home with aerosol and gauze soaked with silver sulfadiazine, vitamin lidocaine
5/10/2021	Wound with good evolution. Lesion area: 22.31 cm^2^; perimeter: 23.67 cm; necrotic tissue area: 14.07 %; Granulation area percentage: 85.85 %
5/10/2021	Amputation stump ulcer with fibrinogranulating background, a notable decrease in diameter, patient reports no pain even at the time of healing. Continuous home treatment with aerosol and gauze soaked with silver sulfadiazine, lidocaine, vitamin A
8/11/2021	Good evolution, currently without pain without antibiotic therapy and decrease in diameter, continues with cures at home with aerosol and gauze soaked with silver sulfadiazine, lidocaine, vitamin A
8/11/2021	Wound with good evolution. Lesion area: 18.015 cm^2^; perimeter: 28.319 cm; necrotic tissue area %: 1.729; Granulation area percentage: 96.0 %
21/12/2021	Ulcer instead of amputation stump, with good evolution lesion area 1.18 cm^2^, perimeter 7.623 cm, necrotic tissue area 1.159 %, granulation percentage 98.2 %

The ulcer progressed to the fourth, third, and second fingers, so it was decided to perform a transmetatarsal amputation on April 26, 2021. The surgery took place at Polo Sanitario Malvinas Argentinas, a hospital in the Province of Buenos Aires, Argentina. The traumatology team treating the diabetic foot case directed the patient to the operating room for a digital amputation procedure. This occurred amid the COVID-19 pandemic, when only emergency procedures were allowed. In the intraoperative phase of the procedure, it was noticed that the hallux had a cyanotic discoloration and that the metatarsal bones heads were infected, so the surgical team opted for a transmetatarsal amputation.

Postsurgical follow-up showed poor healing evolution, with necrotic edges and slough. Several treatments were tried on the affected area for 3 months, with no wound improvement.

In line with the usual treatment for this type of cases, mechanical debridement was performed on the wound once or twice a week. Initially, iodoform gauzes were employed for mechanical debridement, but this approach proved ineffective in yielding favorable results and managing biofilm adequately. The wound was also treated with silver alginate and collagenase, which were the available options during the COVID-19 pandemic, and which also failed to promote healing.

Seeing the poor evolution, the patient's characteristics and the wound contamination, on June 22, 2021, a new treatment was prescribed: gauze dressings soaked in silver sulfadiazine, lidocaine, and vitamin A (Platsul-A® gauze dressings, Soubeiran Chobet SRL, Argentina) and silver sulfadiazine, lidocaine, and vitamin A aerosol (Platsul-A® aerosol, Soubeiran Chobet SRL, Argentina). A.

During the inflammation stage of wound healing, the product was applied every 12 h.

The patient was instructed to clean the wound at his home, applying Platsul-A® aerosol after drying the wound and covering it with Platsul-A® gauze dressings and a sterile dressing twice per day.

Wound evolution was monitored through periodical photographs. Using the ImageJ software from the National Institutes of Health (NIH), USA, data were collected regarding surface, perimeter, the maximum distance between the edges, necrotic tissue and slough area, percentage of granulation tissue and tridimensional images of the wound bed.

## 3 Results

The treatment achieved a remarkable wound evolution, significantly reducing the necrotic tissue, promoting the growth of granulation tissue, and reducing the size of the wound. Moreover, the infection did not progress, and the coaptation of the wound edges was accomplished. The patient did not report any issues during treatment.

We do not rule out Marjolin's ulcer as a diagnosis. Still, it should be borne in mind that, given the constraints imposed by the COVID-19 pandemic, our choice for alternative diagnoses and treatments were significantly limited. For instance, other types of advanced healing dressings were not available to us at that moment.

Although the patient was initially scheduled for digital amputation surgery, a change in the surgical plan occurred when, in the operating room, it was noticed that the hallux had a cyanotic discoloration and that the metatarsal bones heads were infected. Consequently, a decision was made to opt for a transmetatarsal amputation. Given the moderate-to-acute artery disease and the bone infection, a bone biopsy was performed on the remaining bone, testing positive for *Pseudomonas aeruginosa*, *Enterococcus faecalis*, and *Escherichia coli*. This shift in the surgical approach represented the optimal course of action to prevent further amputation and safeguard the patient's quality of life.

**Fig. 1:**
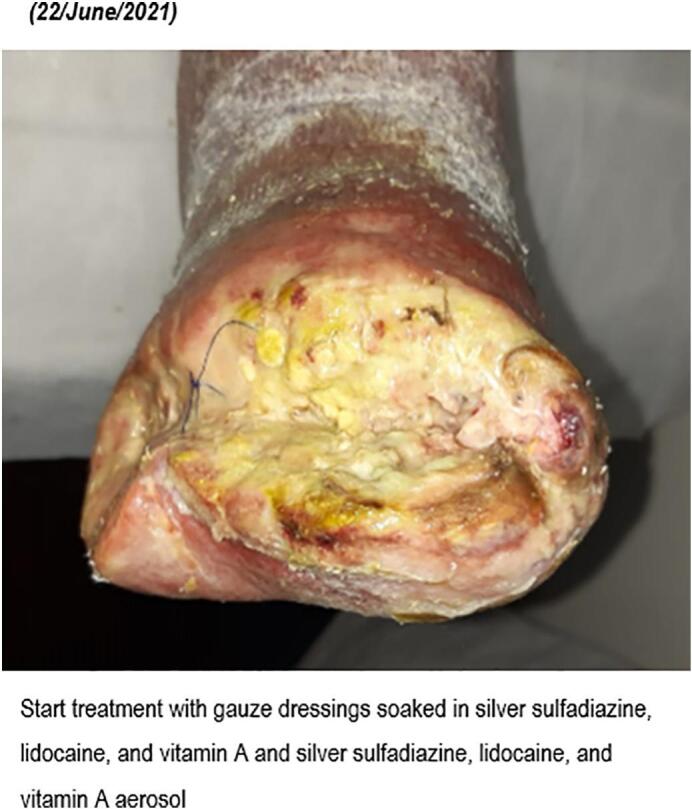
Wound at the beginning of treatment (22/June/2021).

**Fig. 2:**
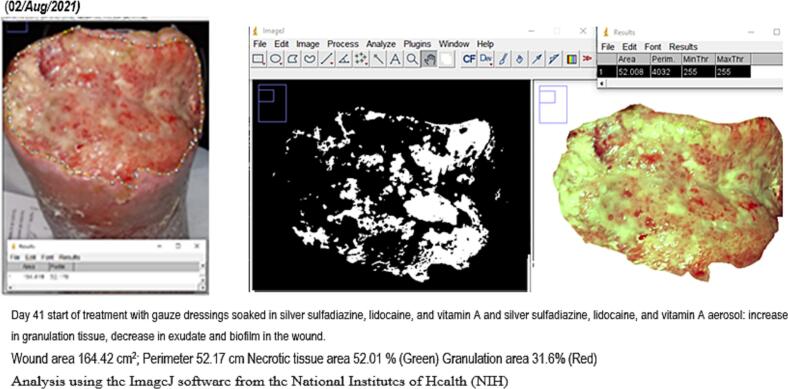
Slough area, perimeter and percentage determination (02/Aug/2021).

**Fig. 3:**
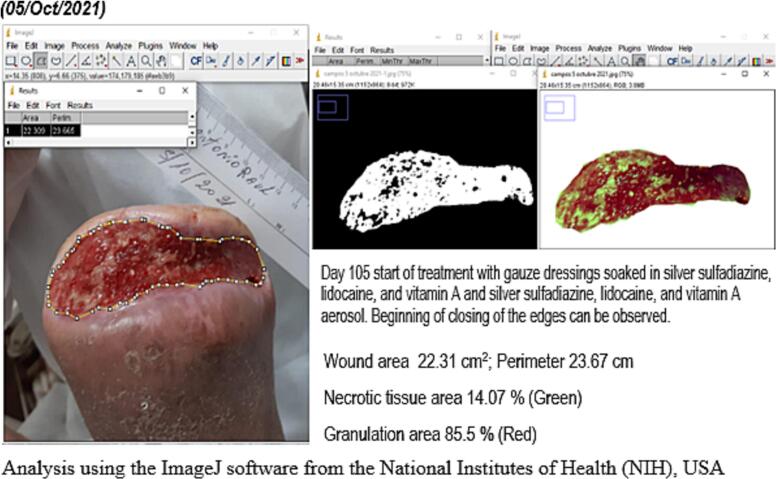
Slough area, perimeter and percentage determination (05/Oct/2021).

**Fig. 4:**
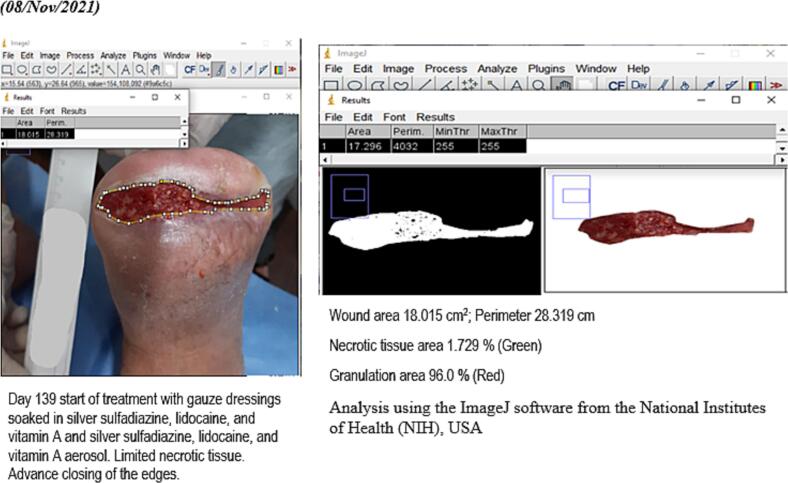
Slough area, perimeter and percentage determination (08/Nov/2021).

**Fig. 5:**
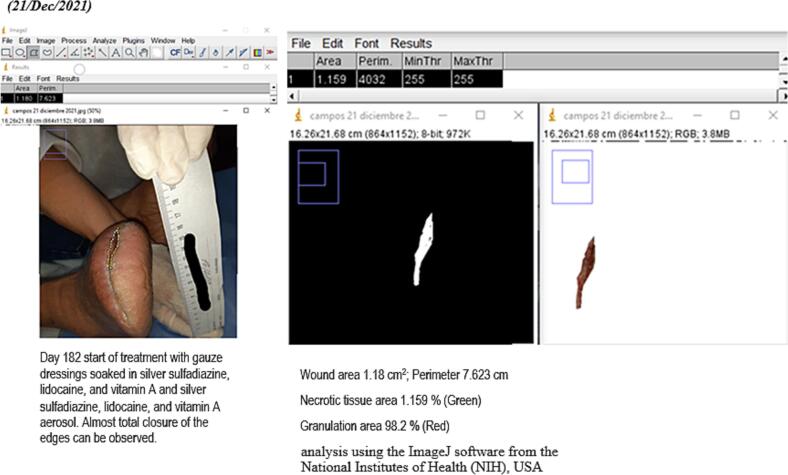
Slough area, perimeter and percentage determination (21/Dec/2021).

In approximately 4 months, the wound area and perimeter shrunk from 164.42 cm^2^ to 1.18 cm^2^ and from 52.17 cm to 7.623 cm, respectively (Table 3, Fig. 1, Fig. 2, Fig. 3, Fig. 4, Fig. 5). Necrotic tissue went from 52.01 % to 1.159 % in 141 days. In that same period, the granulation tissue grew from 31.6 % to 98.2 % (Table 3, Fig. 1, Fig. 2, Fig. 3, Fig. 4, Fig. 5). The maximum distance between the edges went from 12 cm to 0.487 cm in 141 days (Table 4, Fig. 1, Fig. 2, Fig. 3, Fig. 4, Fig. 5).

**Table 3:** Wound evolution.

	2/Aug/2021	5/Oct/2021	8/Nov/2021	21/Dec/2021
Wound area (cm^2^)	164.42	22.31	18.015	1.18
Perimeter (cm)	52.17	23.67	28.319	7.623
Necrotic tissue area (%)	52.01	14.07	1.729	1.159
Granulation area (%)	31.6	85.5	96.0	98.2

**Table 4:** Edge distance evolution.

Day	Edge distance (cm)
0	12
64	3
98	2
141	0.487

In our experience, the key factor to take into account when examining a wound is not its diameter but its depth and the infection stemming from the underlying artery or non-artery disease. It also needs to be considered whether the diabetic foot is infected, edematous, mixed, or ischemic.

**Graph 1:**
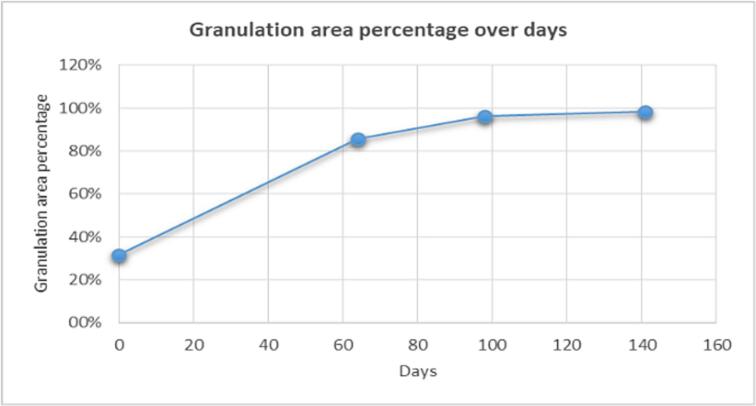
Granulation area percentage over days.

The highest granulation rate was observed on the first 64 days at 0.84 % per day. On the next 34 days, granulation was 0.31 % per day, and in the final stage of the treatment, it was 0.05 % per day (Graph 1). The greatest reduction of the wound size and approximation of the edges was observed in the first 64 days.

**Graph 2:**
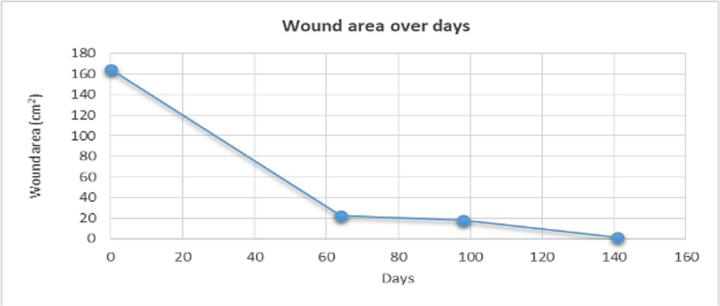
Wound area over days.

**Graph 3:**
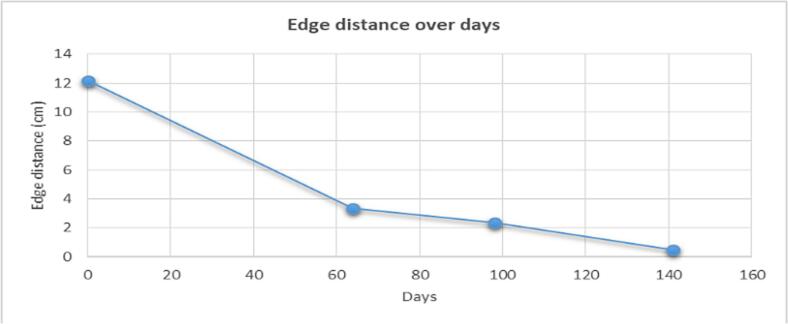
Edge distance over days.

Based on the wound surface measurements, there was a 2.22-cm^2^/day reduction rate on the first 64 days, a 0.126-cm^2^/day reduction rate on the next 34 days and a 0.39-cm^2^/day reduction rate in the final stage of the treatment (Graph 2). Based on the edge distance measurement, there was a 0.14-cm/day approximation rate on the first 64 days, a 0.029 cm/day approximation rate on the next 34 days and a 0.035 cm/day approximation rate in the final stage of the treatment (Table 4, Graph 3).

**Fig. 6:**
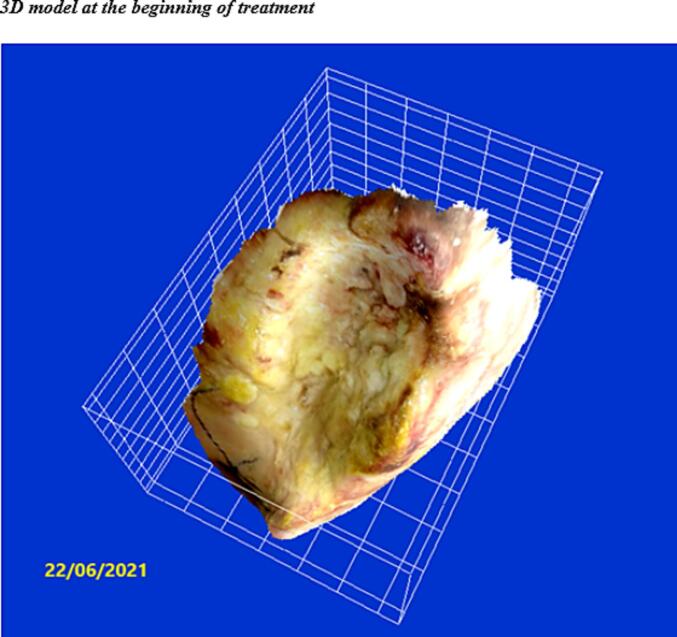
3d model at the beginning of treatment.

The 3D model shows a progression toward a regular, flat wound surface, with a significant approximation of the edges and a considerable reduction in size (Fig. 6). The visual assessment shows that the stump is completely covered with dermal tissue and that the skin is adequately tense. The scar is strategically located and causes no discomfort to the patient.

No adverse effects were reported during treatment.

## 4 Patient perspective

Before starting treatment, the patient reports very little expectation. He was about to sell his truck and had stopped working as a bricklayer. He was in a wheelchair and manifested himself as very depressed and concerned about the lack of efficacy of treatments. He was in a lot of pain and, seeing his wound, assumed that he would lose his leg. He manifests a great depression.

When starting treatment, he reports that the pain subsided. He enthusiastically recounts the improvement of the wound. He noted a change in his mood and improved expectations. He did not sell his truck and reported that he will go back to work as a bricklayer and mason. Today he tells us that he has a normal life thanks to the saving of his leg. He is not depressed.

## 5 Discussion

Among the complications that could appear after a transmetatarsal amputation caused by a diabetic foot, infection is the main factor that may undermine adequate wound healing and pose a higher risk of further amputation. Therefore, adequate wound handling – including surgical debridement of the wound bed, targeted antibiotic treatment, and topical treatment – is paramount to achieving a good outcome. Different types of bacteria have been isolated in different countries and continents. In Latin America, there is a high prevalence of Gram-negative microorganisms, both in soft tissue an in bones, even in what the Infectious Diseases Society of America (IDSA) classifies as mild infections. However, Gram-positive *Staphylococcus aureus* is still the most prevalent microorganism, followed by Gram-negative *Pseudomonas aeruginosa*. Considering that Gram-negative organisms have high resistance to oral antibiotics and that the likelihood of infection increases when the wound takes longer to heal, topical treatment to close the wound becomes a priority [7].

In a recent local study on diabetic foot patients [8], findings show a high rate of major amputation, a low healing rate (53 %) even with minor amputations, and a 35.1 % wound persistence rate.

In our case, the wound was not healing adequately. A.

In this case, what prevented healing was not the eschar but the presence of biofilm and exudate in the wound area, which were proving difficult to manage.

As the biofilm on the wound was preventing healing and as there was no viable granulation tissue even after mechanical debridement, it was decided to treat the wound with the silver sulfadiazine spray, which promotes biofilm elimination.

However, a notable breakthrough emerged with the introduction of treatment involving silver sulfadiazine, lidocaine, and vitamin A, showcasing efficacy in addressing wounds associated with a mixed diabetic foot.

To assess diabetic foot infections, we employ the Saint Elian wound score system, which provides a three-tier classification that assists in predicting the optimal course of treatment. This case posed significant challenges— the mixed diabetic foot, originally assessed at grade 3 on the scale, was infected, had no revascularization option and had failed to improve following prior treatment.

The application of silver sulfadiazine, lidocaine, and vitamin A demonstrated significant improvements in wound evolution and exudate management. Additionally, there was a marked reduction in the pain experienced in the wound area.

The gauze dressings soaked in silver sulfadiazine, lidocaine and vitamin A were changed every 12 h. It is worth noticing that the patient did not report any pain or inconveniences during wound tending or at any point of the treatment. As lidocaine is part of a cream embedded on a cotton mesh, it does not come into contact with the wounded tissue immediately. In addition, the concentration of lidocaine in the products is 0.666 %, which is too low to notice any systemic effects.

None of the effects of the drugs contained in the products used to treat this patient were inhibited due to the interaction with the other drugs. On the contrary, our results indicate that treating wounds with a combination of silver sulfadiazine, lidocaine and vitamin A proves beneficial for patients, which is why we would suggest that this protocol be used in a wider population and that a multicentric study of its application be performed.

Encouraged by these positive outcomes, we expanded the use of the silver sulfadiazine, lidocaine, and vitamin A treatment to address diabetic foot wounds and chronic venous ulcers, treating around 40 patients daily. We continue to collect data for further studies, aiming to obtain comparative statistics on the management of diabetic feet in our patient population.

There was a large percentage of necrotic tissue, humidity, and slough, which indicated that the previous treatments had failed to prevent infection and biofilm formation. It was then decided to use gauze dressings soaked in silver sulfadiazine, lidocaine, and vitamin A plus silver sulfadiazine, lidocaine, and vitamin A aerosol.

Both the gauzes and the spray used to treat the patient contain silver sulfadiazine, lidocaine, and vitamin A, each serving a distinct purpose in the wound healing process. To begin with, silver sulfadiazine has antimicrobial properties, as it damages the bacterial cell wall and disrupts the bacterial DNA structure, precluding its replication. Notably, several gram-negative germs are sensitive to silver sulfadiazine, including *Pseudomonas aeruginosa* (pyocyanic bacteria), *Klebsiella aerogenes* (*Enterobacter aerogenes*), *Klebsiella pneumoniae* (Friedländer's bacillus or Neumann's bacillus), and *Staphylococcus aureus*. In turn, lidocaine is a local anesthetic that acts on the cell membrane. Finally, vitamin A contributes to the induction and control of epithelial differentiation, promoting re-epithelialization. Once applied topically, vitamin A can be absorbed—it is metabolized through oxidation, forming retinoic acid, or tretinoin, in the liver, where vitamin A and retinoic acid conjugate with glucuronic acid.

This treatment was preferred to other alternatives due to several factors. To begin with, the wound area had failed to improve after being subjected to surgical debridement and treated with iodoform gauzes, silver alginate and collagenase. In addition, treatment with vitamin D was deemed unsuitable, as the wound was a deep ulcer with slough. As for 0.5 % mafenide acetate, this local antibiotic—which needs to be applied every 12 h due to its short half-life—has a broad bacteriostatic action against gram-negative aerobic bacilli and anaerobes, but not against yeast. Other disadvantages of using mafenide acetate include that if may lead to metabolic acidosis, thus delaying healing, and that, while it penetrates eschar much more effectively than other topical agents, it causes pain on application. Finally, it is worth highlighting that, at the time when the patient was treated during the COVID-19 pandemic, this antibiotic was not available in Argentina.

At that moment, alternative treatment options, such as skin grafts, were not available at the hospital due to the COVID-19 pandemic. Besides, as the patient's artery disease had no possibility of revascularization, it was not viable to use a skin graft.

As a result, both the wound area and the necrotic tissue quickly shrunk and the granulation tissue increased, and the distance between the edges reduced. No wound edge maceration was observed at any point during treatment.

It is impossible to predict how long a wound will take to heal, as this hinges on the unique assessment of each wound and the underlying pathology of the individual patient. In this case, the patient had a mixed diabetic foot that was hard to treat, as it was both infected and vascularized. In addition, the patient's acute artery disease, associated with the presence of resistant biofilm in the wound area, was the main factor behind the delays in the healing process.

Biofilm is linked to slower wound healing and wound chronicity, as this community of microorganisms in the wound slows down healing even when there are no apparent signs of infection. Therefore, treatment should be geared toward preventing contamination from leading to biofilm formation.

Deep dermal tissue in all chronic wounds hosts multiple bacteria species, most frequently *Pseudomonas aeruginosa* and *Staphylococcus aureus*, both methicillin-resistant (MRSA) and methicillin-susceptible (MSSA) [9]. Other gram-negative bacilli of the *Enterobacteriaceae* family, anaerobic bacteria such as *Clostridium*, yeasts such as *Candida albans*, and fungi such as *Aspergillus* can also be found.

Recent studies [10,11] have described a strong antimicrobial effect of silver sulfadiazine, lidocaine, and vitamin A aerosol against biofilm-forming “superbacteria”, such as *Pseudomonas aeruginosa*, MRSA, and MSSA. Furthermore, topical application of silver sulfadiazine, lidocaine, and vitamin A improves postsurgical scars, preventing infection, reducing pain, and promoting healing [12]. Thus, we consider that the silver sulfadiazine, lidocaine, and vitamin A aerosol was a key factor in healing. Also, the silver sulfadiazine, lidocaine, and vitamin A gauze dressings offered a mechanical barrier to environmental infections.

Measuring the evolution of the wound area is one of the best ways to assess the healing process. In this case, the greatest percentages in wound area reduction, granulation area increase, and edge distance reduction were observed on the first 64 days of treatment. It should be noted that our patient had arterial disease, ischemia, and no revascularization option, which meant that his likelihood of healing was lower than in non-ischemic patients [13,14]. We can then infer that this faster healing is the result of the elimination of biofilm, which, as we already mentioned, is linked to wound chronicity.

**Fig. 7:**
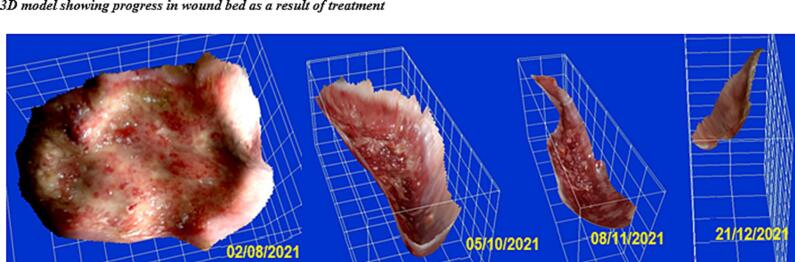
3d model showing progress in wound bed as a result of treatment.

Although at first, the wound edges had slough and were hypertrophic, necrotic, and macerated, treatment quickly reversed this situation. The evolution of the wound is also shown in the 3D images. At the beginning of the new treatment, the edges were not defined, and the surface was rather irregular, with considerable invagination and tunneling and the presence of cavities and fistulas, which typically lead to contamination and persistent infection. At the end of the treatment, however, we could see a remarkable evolution: the surface was smooth, the wound bed was smaller, and the edges were well-defined (Fig. 6, Fig. 7).

After amputation, wound healing needs to be aimed at obtaining a functional, good-quality stump. Besides being painless, the stump should have certain physiological characteristics, such as conical shape, good circulation, adequate mobility, and muscular strength. Skin tension should be neither too tense nor too loose. Fast healing of the wound and approximation of the edges results in faster rehabilitation, thus increasing patients' quality of life. It is noteworthy that vitamin A promotes fast epithelization and increases the number of macrophages in the wound. In addition, it acts as an antioxidant and promotes fibroblast differentiation and collagen synthesis [15]. In this case, the stump achieved all the desirable characteristics, especially regarding skin tension, which suggests that the vitamin A contained in both products significantly improves healing and helps create an optimal stump.

The patient did not report any pain or inconveniences during wound tending or at any point of the treatment. Both products contain 0.66 % lidocaine, a local anesthetic that is broadly used to manage pain. This concentration was enough to avoid the use of systemic analgesics.

## 6 Conclusion

In this clinical case, combined topical use of silver sulfadiazine, lidocaine, and vitamin A aerosol and gauze dressings proved effective in reversing complications and lack of healing in a patient with a transmetatarsal amputation caused by a diabetic foot, besides helping achieve a functional stump that improved the patient's quality of life. As the results show, the infection was quickly contained, the granulation tissue increased, and the wound healed. As this was a senior patient with comorbidities, no revascularization option and a large wound, treating him was a true challenge that required not only surgical treatment but also advanced, tailored wound care and targeted antibiotic treatment prescribed after a biopsy.

All in all, we can conclude that the combined topical use of silver sulfadiazine, lidocaine,and vitamin A aerosol and gauze dressings achieved infection and biofilm control and promoted a quick and effective advanced wound healing in a patient with a transmetatarsal amputation caused by a diabetic foot with a high risk of greater amputation.

Further prospective follow-up studies of postsurgical wound beds are necessary for a more in-depth analysis of how biofilm affects healing in patients with transmetatarsal amputation caused by severe diabetic foot infection.

## Ethical approval

The study is exempt from ethical approval. Because it is a therapy commonly used in our institution for the treatment of wounds.

## Funding

There is no funding or sponsors of the case.

## Author contribution

Saurral R: Wound healing, patient care and article writing.

Pool R, Serrudo M V, Vaisman A, Garibay L , Wound healing and patient care.

Carrió LM: Director of the Service and article writing.

## Guarantor

Carrió LM

## Research registration number

Does not apply.

## Conflict of interest statement

No author declares conflict of interest.
